# Implant survival of cemented arthroplasty following failed fixation of proximal femoral fractures in patients aged 30–60 years: a retrospective study with a median follow-up of 10 years

**DOI:** 10.1186/s12891-022-05587-0

**Published:** 2022-07-04

**Authors:** Mingliang Yu, Minji Yu, Yaodong Zhang, Huihui Cheng, Xianshang Zeng, Si Li, Weiguang Yu

**Affiliations:** 1 Department of Anesthesiology, Wuhan Fourth Hospital, No.473, Hanzheng Street, Qiaokou District, Wuhan, 430033 China; 2 Department of Traditional Chinese Medicine, The First Affiliated Hospital, Sun Yat-sen University, No. 58, Zhongshan 2nd Road, Yuexiu District, Guangzhou, 510080 China; 3 Department of Anesthesiology, The First Affiliated Hospital, Sun Yat-sen University, No. 58, Zhongshan 2nd Road, Yuexiu District, Guangzhou, 510080 China; 4 Department of Anaesthesiology, Renmin Hospital of Wuhan University, Wuhan, 430060 Hubei China; 5 Department of Orthopaedics, The First Affiliated Hospital, Sun Yat-sen University, No. 58, Zhongshan 2nd Road, Yuexiu District, Guangzhou, 510080 China

**Keywords:** Survival, Fracture, Arthroplasty, Fixation, Revision

## Abstract

**Background:**

Given the unremitting growth in the volume of failed fixations of proximal femoral fractures (PFFs) in recent years, it is predictable that total hip replacements (THRs) will be the preferred surgical procedure. The long-term survival of cemented THR (CTHR) revisions remains controversial in patients aged 30–60 years. The goal of this retrospective review was to evaluate the 10-year survival of CTHRs following prior failed primary fixations of PFFs in patients aged 30–60 years.

**Methods:**

We retrospectively identified CTHR revisions implemented at four medical centres during 2008–2017 for a failed primary fixation of PFFs in consecutive patients aged 30–60 years. The primary endpoint was implant survival calculated using the Kaplan–Meier method with 95% confidence intervals (CIs); secondary endpoints included functional scores assessed by Harris hip scores (HHS) and main revision-related orthopaedic complications. Follow-up was executed at 1, 2, 3, and 8 years following revision and then at 1-year intervals until the revision, death, or study deadline, whichever occurred first.

**Results:**

In total, 120 patients (120 hips) who met the eligibility criteria were eligible for follow-up. The median follow-up was 10.2 years (range, 8–12 years). Kaplan–Meier survivorship showed that implant survival with revision for any reason as the endpoint was 95% at 5 years (CI: 93–97%), 89% at 8 years (CI: 86–92%), and 86% at 10 years (CI: 83–89%). Patients treated with three hollow screws had better revision-free survival than patients treated with proximal femoral nail antirotation (PFNA), dynamic hip screw (DHS) or titanium plate plus screws (three *p* < 0.05). Functional scores were apt to decrease gradually, and at the final follow-up, the mean HHS was 76.9 (range, 67.4–86.4). The overall rate of main revision-related orthopaedic complications was 18.3% (22/120).

**Conclusion:**

CTHR implemented following prior failed primary fixations of PFFs tends to afford an acceptable 10-year survival, along with advantageous HHS and a low rate of main revision-related orthopaedic complications, which may support an inclination to follow the utilisation of CTHRs, especially in revision settings for intracapsular fractures.

## Background

Failed fixations of proximal femoral fractures (PFFs) are infrequent but distressing and incapacitating complications and may be frequently accompanied by persistent pain, dysfunction, poor quality of life, and even life threatening [1–3]. With the expansion of surgical indications for total hip replacements (THRs) [4], it is expected to increase the demand for THRs, which are a universal and reliable procedure characteristically executed for the reduction of hip symptoms and have been approved as a surgical protocol for converting a failed fixation of PFFs with the aim of relieving pain, strengthening stability, and restoring function [5, 6]. Despite improved hip function attributed to THRs, prior studies [7, 8] have indicated that approximately 15% of patients have to deal with this dilemma of component loosening that may have unfavorable kinematic aftereffects since mechanically aligned components may be passively shifted and trigger a variation in the angle of the femur, which can eventually lead to premature failure of THRs. Furthermore, available data [9, 10] indicate that component loosening is commonly regarded as a catastrophic failure of THRs owing, to some extent, to concern over reduced component survival. The ever-increasing rate of component loosening related to uncemented or hybrid THRs may be driven not only by different designs or brands of implants but also by poor surgical experiences attributed to frequently updated THR products [11] and has received increasing concern from clinical surgeons, particularly when the prosthesis is improperly positioned or the occurrence of pericomponent fractures prompted revision THRs to be implemented [12, 13].

As with other potential mechanisms associated with loosening-resistant prostheses, cemented THRs (CTHRs) may have positive effects on hip rehabilitation while re-establishing hip mechanics [14]. Prior studies [15, 16] have indicated that CTHRs have substantial advantages with respect to implant survival, functional status, and rapid rehabilitation in patients with specific age spans. Nonetheless, these studies assessing the effect of CTHRs on clinical results, particularly on implant survival, have been subject to small sample size, large age span, short-term follow-up, and diverse definitions of implant failure, which greatly impacts the elucidation of their findings. With the increase in the contributing baseline variables affecting the survival of CTHRs and the burden of revision CTHRs liable to rise at a great rate, there remains an urgent need to provide clinical practice-based evidence on the assessment of CTHR survival, as concerns about implant loosening have not been highlighted in cases where cement-triggered osteolysis may be associated with component loosening [17, 18]. How to make a more advantageous choice and how to minimise hip dysfunction in patients who play a major economic role in the family may be a priority and urgent issue.

To date, there are few analyses assessing survival trends over time and outcomes associated with CTHR revisions. Furthermore, literature on the long-term survival of the conversion of the failed fixations of PFFs to a single brand of CTHRs in patients aged 30–60 years, irrespective of subgroup analysis of age, remains lacking. Hence, this study aim was to retrospectively evaluate the 10-year survival of CTHRs following prior failed primary fixations of PFFs in patients aged 30–60 years.

## Methods

### Study population

A retrospective cohort of consecutive patients aged 30–60 years undergoing CTHR revision following a failed primary fixation of PFFs from September 2008 until December 2017 was identified from Joint Surgery Centres, The First Affiliated Hospital of Sun Yat-sen University and Wuhan Fourth Hospital. CTHR was implanted for the recommended indications, as reported by Mahmoud et al. [19]. The indications for failed primary fixations of PFFs involved varus collapse of the femoral head, cut-out, femoral shaft fracture, and nonunion. The indications for revision CTHRs involved component malposition, component loosening, repeated dislocation, pericomponent fractures, infection, and osteolysis. CTHR surgery was implemented per standard technique, as described in our published study [20]. Third-generation cementing techniques were used in all patients included in this study. Details of the CTHRs used in this study are presented in Table 1. The postoperative protocols were consistent with our earlier reports [20]. The assessment of overall comorbidity burden was implemented using the Deyo adaptation of the Charlson comorbidity index (CCI). Patients who had the following conditions were excluded from this study: uncertain patient characteristics (e.g., with or without autogenous iliac bone graft, Harris Hip Scores (HHS) records, CCI, body mass index [BMI], and bone mineral density [BMD]), allogeneic bone implantation, bilateral or staged CTHR procedures, conversion triggered by periprosthetic joint infection, failure to ambulate on their own, osteomyelitis-associated osteolysis, clinical evidence of neuromuscular conduction disorder (e.g., myasthenia gravis), spinal disorders secondary to tuberculosis, virus, and trauma, cerebrovascular accidents, acetabulum deformity, malignant tumour, tonic or progressive muscular dystrophy, and mental disturbance (e.g., Alzheimer’s disease).

**Table 1:** Details of the CTHRs used in this study

	Stem	Cup	Cement type	Head
CTHR(*n* = 120)^a^	cemented polished tapered stem	uncemented Trabecular Metal monoblock cup	antibiotic-impregnated cement	(28, 32, or 36 mm)

*CTHR* cemented total hip replacement

^a^Zimmer, Warsaw, Indiana

### Outcomes and variables

Patient demographics (age, sex, BMI, BMD]), revision information, and implant survival data pertaining to CTHRs were collected. The key intervention variable was CTHR revision. The primary endpoint was the implant survival estimated by the Kaplan–Meier survival. Implant survival was calculated from the date of CTHR revision until the date of revision CTHRs. The definition of revision was removal of one or more components, which was consistent with prior studies [4, 8, 10]. The secondary endpoints included functional scores measured using Harris hip scores (HHS) and main revision-related orthopaedic complications (component malposition, component loosening, repeated dislocation, pericomponent fractures, infection, and osteolysis). The definition of component loosening was consistent with our published paper [20]. Follow-up was executed at 1, 2, 3, and 8 years following revision and then at 1-year intervals until the revision, death, or study deadline, whichever occurred first.

### Statistical analysis

We analysed the baseline data and prevalence of comorbidities that may have an effect on the results for patients aged 30–60 years experiencing a conversion of prior failed primary fixations of PFFs to CTHRs. Analysis was executed with revision for any reason as the primary endpoint and with component malposition, component loosening, repeated dislocation, pericomponent fractures, infection, and osteolysis as a secondary endpoint. All analyses were executed separately for each endpoint. We applied graphical methods to plot linear trends in functional outcomes. For continuous data, the median or mean was embodied. Median follow-up was estimated by the reversed Kaplan–Meier method. Implant survival of CTHRs was calculated with the Kaplan–Meier method with 95% confidence intervals (CIs) and with revision CTHRs for any reason as the endpoint. Kaplan–Meier survival curves were stratified by age (30 ≤, < 45 or 45 ≤, < 60), sex (male or female), autogenous iliac bone graft (yes or no), prosthesis type of prior fixation failures (three hollow screws, proximal femoral nail antirotation[PFNA], dynamic hip screw[DHS], and titanium plate plus screws), time to conversion (< 3 or ≥ 3), femoral head size (standard [28 mm and 32 mm] or custom [36 mm]), CCI at conversion (low or medium or high), indications for CTHR revision (instability or mechanical failure or both), and American Society of Anesthesiologists (ASA) physical status (1 or 2 or 3). Distinctions in survival between subgroups were assessed by the log-rank test. A 2-sided *p* value of < 0.05 was regarded as significant. Data were principally analysed using SAS 9.4 (Cary, NC).

## Results

**Fig. 1:**
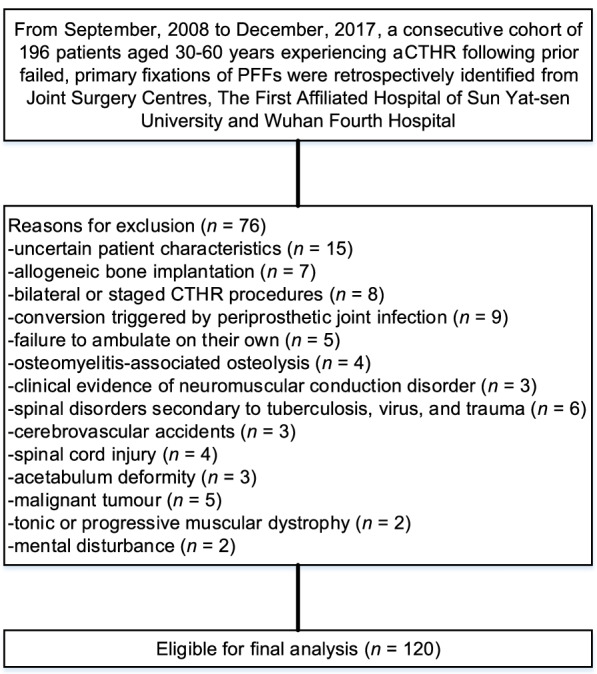
Flow diagram presenting the method for the identification of patients aged 30–60 years to evaluate the 10-year survival of CTHRs following prior failed primary fixations of PFFs

In total, 196 patients aged 30–60 years who experienced a secondary CTHR revision following prior failed primary fixations of PFFs were included. Among them, 76 patients were eliminated in accordance with the current exclusion criteria, and 120 patients (120 hips) who met the eligibility criteria were eligible for follow-up (Fig. 1). There were 67 (55.8%) men and 53 (44.2%) women with a mean age of 54 years (range, 30–60 years), a mean BMI of 26.8 kg/m^2^ (range, 18.3–38.2 kg/m^2^) and a mean BMD of − 3.76 (range, − 2.94 to − 4.58) at the time of CTHR revision. Sixty-three (52.5%) patients underwent an autogenous iliac bone graft during CTHR revision, and 57 (47.5%) did not experience an autogenous iliac bone graft. The prosthesis types of prior fixation failures of PFFs were PFNA in 37.5%, three hollow screws in 30%, DHS in 22.5%, and titanium plate plus screws in 10.0%. The femoral head size used in this study was 28 mm in 35%, 32 mm in 40%, and 36 mm in 25.0%. The most common CCI at conversion was low (49.2%), followed by medium (35.8%). Only 18 (15.0%) of 120 patients had a high CCI at conversion. Indications for CTHR revision were instability in 55.0%, mechanical failure in 29.2%, and both in 15.8%. The mean HHS prior to conversion was 51.5 (range, 37.4–65.6). Table 2 shows the patient characteristics at baseline.

**Table 2:** Patient characteristics at baseline

Variable	CTHR (*n* = 120)
Age (years), No.%	
30 ≤, < 45	45(37.5)
45 ≤, < 60	75(62.5)
Sex, No. %	
Male	67(55.8)
Female	53(44.2)
BMI (kg/m^2^)	
Mean (range)	26.8 (18.3–38.2)
BMD	−3.76 ± 0.82
Autogenous iliac bone grafts, No.%	
Yes	63(52.5)
No	57(47.5)
Type of prior fractures, No.%	
Femoral neck fractures	
Subcapital	12(10.0)
Transcervical	24(20.0)
Base neck	19(15.8)
Intertrochanteric fractures (Evans-Jensen classification)	
Type I	8(6.7)
Type II	13(10.8)
Type III	21(17.5)
Type IV	18(15.0)
Type V	5(4.2)
Prosthesis types of prior fixation failures, No.%	
PFNA^a^ (femoral neck fractures and intertrochanteric fractures)	
Base neck	19(15.8)
Type I	2(1.6)
Type II	3(2.5)
Type III	10(8.3)
Type IV	8(6.6)
Type V	3(2.5)
Three hollow screws^b^ (femoral neck fractures)	
Subcapital	12(10.0)
Transcervical	24(20.0)
DHS^b^ (intertrochanteric fractures)	
Type I	1(0.8)
Type II	3(2.5)
Type III	11(9.1)
Type IV	10(8.3)
Type V	2(1.6)
Titanium plate^c^ plus screws^b^ (intertrochanteric fractures)	
Type I	5(4.2)
Type II	7(5.8)
Time to conversion (mos), No.%	
< 3	78(65.0)
≥ 3	42(35.0)
Femoral head size (mm), No.%	
28	42(35.0)
32	48(40.0)
36	30(25.0)
CCI at conversion, No. %	
Low	59(49.2)
Medium	43(35.8)
High	18(15.0)
Indications for CTHR revision, No. %	
Instability	66(55.0)
Mechanical failure	35(29.2)
Both	19(15.8)
ASA physical status, No.%	
1	35(29.2)
2	63(52.5)
3	22(18.3)
HHS prior to conversion	51.5 ± 14.1
Follow-up (years)	
Median (range)	10.2(8–12)

*CTHR* cemented total hip replacement, *BMI* body mass index, *BMD* bone mineral density, *PFNA* proximal femoral nail antirotation, *DHS* dynamic hip screw, *CCI* Charlson comorbidity index, *ASA* American Society of Anesthesiologists, *HHS* Harris hip scores

^a^Smith & Nephew, Memphis, Tennessee

^b^Double Medical, Xiamen, China

^c^Synthes, West Chester, PA, USA

### Primary endpoint

**Fig. 2:**
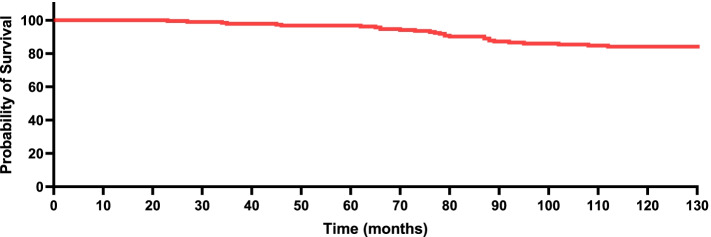
Kaplan–Meier survival curve with revision for any reason as the endpoint

**Fig. 3:**
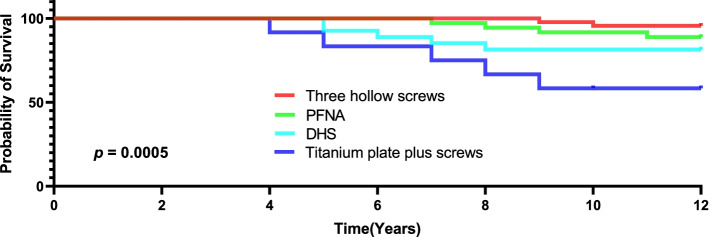
Kaplan-Meier survival curve for each implant with revision for any reason as the endpoint

The median follow-up was 10.2 years (range, 8–12 years). In total, 13.3% (16 CTHRs) of patients experienced a revision CTHR during the study period, of which 3 patients treated with titanium plate plus screws were due to component malposition, 4 PFNA-treated patients and 4 DHS-treated patients were due to stem loosening, 2 patients treated with three hollow screws were due to stem loosening, one patient treated with a titanium plate plus screws was due to repeated dislocation, one DHS-treated patient was due to stem fractures, and one patient treated with a titanium plate plus screws was due to infection and osteolysis. Implant survival was 95% at 5 years (CI: 93–97%), 89% at 8 years (CI: 86–92%), and 86% at 10 years (CI: 83–89%), as shown in Fig. 2. Figure 3 shows the subgroup survival results. Patients treated with three hollow screws had better revision-free survival than patients treated with PFNA, DHS or titanium plate plus screws (three *p* < 0.05). Patients experiencing PFNA had better revision-free survival than patients experiencing DHS or titanium plate plus screws (both *p* < 0.05). Patients experiencing DHS had better revision-free survival than patients experiencing titanium plate plus screws (*p* < 0.05). Substantial distinctions in implant survival were also detected with regard to autogenous iliac bone grafts, prosthesis types of prior fixation failures, time to conversion, CCI at conversion, and indications for CTHR revision (all *p* < 0.05). No noteworthy differences in implant survival were observed with respect to sex, femoral head size, or ASA physical status (all *p* > 0.05).

### Secondary endpoints

**Fig. 4:**
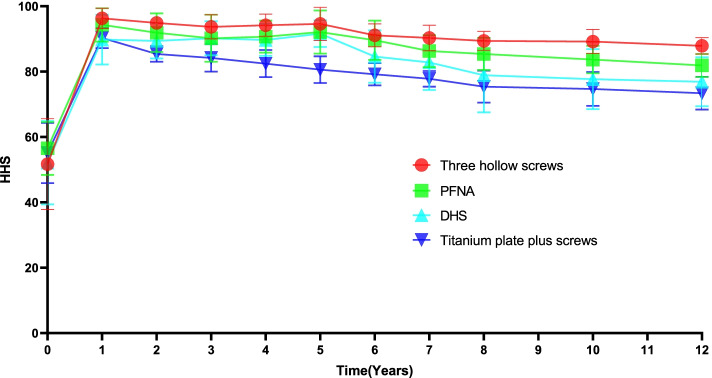
The variation trend of HHS after CTHR revision

Functional scores are shown in Table 3. The trend of functional scores after CTHR revision for each failed implant with the extension of follow-up time is shown in Fig. 4. The functional scores of each implant peaked at the first year after CTHR revision and were apt to decrease gradually from the 5th year after CTHR revision. It was expected that it may be further reduced with the extension of the follow-up period. For the entire study population, the mean HHS was 76.9 (range, 67.4–86.4) at the final follow-up. Patients experiencing three hollow screws had better HHS than patients experiencing PFNA, DHS or titanium plate plus screws. Table 4 shows the main revision-related orthopaedic complications in patients undergoing CTHR revision. In the present study, 6 (5.0%) patients were affected by component malposition, 15 (12.5%) suffered stem loosening, 3 (2.5%) had repeated dislocation, 3 (2.5%) had a stem fracture, and 4 (3.3%) had infection and osteolysis. Of the 15 patients with stem loosening, 10 patients with intertrochanteric fractures were treated with DHS, and 5 patients with femoral neck fractures were treated with PFNA (*p* = 0.301). Among 120 patients, 22 patients had 31 orthopaedic complications. The overall rate of main revision-related orthopaedic complications was 18.3% (22/120).

**Table 3:** Functional scores of patients aged 30–60 years undergoing CTHR revision

Year(s) after CTHR revision	CTHR (*n* = 120)
1	88.3 ± 8.1
2	89.4 ± 7.4
3	90.7 ± 6.7
4	91.2 ± 6.4
5	93.1 ± 6.6
6	85.6 ± 9.0
7	82.8 ± 11.4
8	80.1 ± 12.2
10	78.2 ± 10.7
12	76.9 ± 9.5

*CTHR* cemented total hip replacement

**Table 4:** Main revision-related orthopaedic complications in patients undergoing a CTHR

Variable, No.%	CTHR (*n* = 120)
Revision	16(13.3)
Component malposition	6(5.0)
Component loosening(stem)	15(12.5)
Repeated dislocation	3(2.5)
Pericomponent fracture (stem)	3(2.5)
Infection and osteolysis	4(3.3)

*CTHR* cemented total hip replacement

## Discussion

This retrospective review of implant survival of CTHRs following prior failed primary fixations of PFFs in patients aged 30–60 years demonstrates that CTHR may be associated with an acceptable 10-year survival, noteworthy improvement of functional status, and a tolerable rate of main revision-related orthopaedic complications. The current findings boost a body of evidence that CTHRs could be conducive to receiving long-term survival benefits, restoring hip function, and allaying the concern about distressing long-term survival, and possibly will be a salvage procedure in patients aged 30–60 years, especially in revision settings for intracapsular fractures, although consensus regarding long-term survival of CTHRs following prior failed primary fixations of PFFs has not been reached [21]. Furthermore, there remains concern [21, 22] that the rate of implant survival may have been overestimated as a result of dissimilar economic levels and tolerance to component failure per patient as well as the retrospective design. Although the introduction of cement-type prostheses has been regarded as a cornerstone for an efficacious and hard-wearing THR [21], a relatively high rate of main revision-related orthopaedic complications was noted in this study, which may be attributed to the fact that CTHR is not a primary surgery but a secondary surgery, and in this setting, massive bone loss and increased bone fragility may explain the unfavourable result.

The present findings were aligned with and extend the findings of prior studies [18, 23], which indicated that primary CTHRs were associated with long-term implant survival, along with good functional results and an acceptable orthopaedic complication. For patients aged 30–60 years with prior failed primary fixations of PFFs, there was no abundant evidence behind the routine use of secondary CTHRs and no efficient outcome evaluation at the prosthesis level [19]. Our findings may provide supplementary data to favor the use of CTHRs for the secondary treatment of PFFs. The 10-year survival reported in the present study may be slightly lower than the survival outcome of primary CTHRs in elderly patients or even in some younger patients. This may be because our study included 65 (54.2%) patients with intertrochanteric fractures. For intertrochanteric fractures, substantial changes in the structure, shape, properties and composition of the proximal femur may result in a decrease in the number of cortical bones and bone trabeculae in the proximal femur and a decrease in compressive strength, and fracture-induced osteocyte degeneration may impair the mechanical integrity of the proximal femur, resulting in loss of strength during osteocyte senescence [24].

The results [25] based on the Swedish Hip Arthroplasty Register showed that CTHRs had a 94% revision-free 10-year survival, a lower risk of revision for any reason (risk ratio[RR] = 1.5, 95% CI: 1.4–1.6) and for component loosening (RR = 1.5, CI: 1.3–1.6). Their conclusions indicated that survival of CTHRs is superior to that of uncemented THRs, which is primarily attributed to better performance of CTHRs. Similarly, a prior study [26] of 373 primary CTHRs showed that the 8-year revision-free survivorship was 94% (CI: 91–96). In 2016, a study [21] of a brand-level comparison of CTHRs assessing implant survival from a multinational database with a large sample size indicated that the 10- and 15-year survival of CTHRs ranged from 85.0 (CI: 80.5–89.5) to 99.0 (98.4–99.6) and 83.9 (82.5–85.3) to 92.6 (91.2–94.0), respectively. In 2010, a retrospective study [23] of 140 consecutive patients aged 40–50 years treated with a primary CTHR showed a 10-year survival of 88% (95% CI: 82–94) with revision for any reason, and survival with aseptic loosening for any reason was 94% (95% CI: 90–99). In 2018, a prospective study [27] of 100 patients (105 hips) aged 55 years or younger undergoing a single CTHR showed that the survivorship at a minimum of 22 years with revision for any reason was 97% (95% CI 95–98). In 2022, a prospective study [28] of 860 patients undergoing a matched CTHR showed that the 14-year overall revision-free survival was 96.0%. Discrepancies between our survival outcomes and those reported in previous studies remain, which may be attributed in part to differences in the patient’s bone and soft tissue conditions [8, 19]. Patients who request CTHR revision commonly suffer from disturbing dilemmas of osteopenia, bone structure disorders, and poor soft tissue conditions, which may have a negative impact on implant survival [18, 21].

Patient age at the time of surgery may be a reference factor, not a determining factor [29, 30]. A study [31] reported that the use of CTHRs could provide limited survival benefits in patients aged less than 60 years over time. Their outcome tends to be restrained by short-term follow-up and a limited number of patients and has failed to be verified by large registration data. Discernment of the necessity for promising evidence-based management is coming into view by establishing a long-term follow-up mechanism [32]. Furthermore, such a formulation may contradict the biology of the osseointegration of CTHRs, where the potential of CTHRs to integrate into host bone has been utilised as a theoretical basis for the superiority of CTHRs, especially given osteoarthritis-related osteoporosis [33]. Hence, we conclude that there is no evidence to support the view that young patients diagnosed with severe osteoporosis have superior long-term revision-free survival after uncemented THRs than after CTHRs. CTHRs may have broad surgical indications and good long-term survival [20, 21], particularly for patients with pathology-related bone (e.g., rheumatoid arthritis, osteoarthritis), irrespective of age, although there remain some defects, such as difficulty in revision and cardiovascular events during surgery [18].

With the increase in the number of CTHR revisions following prior failed primary fixations of PFFs, revision CTHR procedures will probably continue to increase since the factors associated with CTHR failures are multifaceted and difficult to address at the root [20], despite definite improvements in prosthetic materials and designs. The revision CTHR procedure may be a convoluted and backbreaking procedure, predominantly in younger and more active patients who urgently need limb function recovery from injury [8, 20]. Nevertheless, revision CTHR may exacerbate the occurrence of component loosening as a result of the well-established inequality between axial and rotating loads caused by massive bone loss [7, 34]. Component loosening attributed to the combination of implant type and osteolysis (or infection) could lead to the revision of secondary CTHRs [20, 34], although distinctions between primary and secondary CTHRs are still being debated [18, 21, 23]. Several studies [35, 36] have indicated that bone cement contributes to osteolysis. However, convincing data on osteolysis induced by bone cement remain lacking, as prospective large-sample studies are required to confirm this result [36]. Accordingly, concerns about the application of cement-type prostheses remain [37]. Cement-type prostheses were identified as a key contributor to survival results and have since been the focus of component loosening associated with the combination of implant type and osteolysis (or infection) [20, 23]. Despite promising survival benefits derived from the present study assessing the survival of CTHRs, we are unable to advocate the routine use of CTHRs in each individual entailing a THR because the component-bone stabilising impact on survival results among individuals still needs further evaluation.

Several shortcomings should be acknowledged prior to elucidating the current findings. First, a retrospective design has its inherent drawbacks. Some research subjects were excluded according to stringent exclusion criteria, which may fail to reflect actual practice and tend to be burdened by limited external validity. Second, the primary and secondary endpoints are easily restricted by human factors and implant versions. Lack of details in every updated version of CTHRs is a relatively large flaw in this study, as practice-based evidence shows [20] that subtle differences between implant versions may have a significant impact on the survival outcome of the implant. Notably, the lack of partial clinical information may not warrant the perfect validity of the data, as there are always uncontrollable factors that affect the reliability of conclusions. Even so, due to the large time span of our follow-up, this problem seems to be difficult to avoid. Third, a control group was lacking in this study. Given the 10-year follow-up results following CTHR revision, the absence of a control group may have a limited impact on the outcome, although it was impossible to infer causation-related conclusions. Fourth, generalisability is lacking in this study due to the limitations of our study subjects to patients aged 30–60 years.

## Conclusions

This study showed that CTHR fulfilled after prior failed primary fixations of PFFs in patients aged 30–60 years may yield an acceptable 10-year survival and noteworthily improved functional scores and a tolerable rate of main revision-related orthopaedic complications, which may have created an atmosphere for the trend towards increasing utilisation of CTHR, especially in revision settings for intracapsular fractures in patients aged 30–60 years, although our study was retrospectively designed with a large time span of follow-up, lack of information on the implant version, and absence of the control group.

## Data Availability

The datasets generated during and analysed during the current study are not publicly available due to the protection of patient privacy but are available from the corresponding author on reasonable request.
